# Sequelae and Other Conditions in Ebola Virus Disease Survivors, Sierra Leone, 2015

**DOI:** 10.3201/eid2301.160631

**Published:** 2017-01

**Authors:** Hamish Mohammed, Alren O. Vandy, Rebecca Stretch, David Otieno, Mukesh Prajapati, Mauricio Calderon, Mohamed Vandi

**Affiliations:** Public Health England, London, UK (H. Mohammed);; World Health Organization, Kenema, Sierra Leone (H. Mohammed, A.O. Vandy, R. Stretch, D. Otieno, M. Prajapati);; World Health Organization, Freetown, Sierra Leone (M. Calderon);; District Health Management Team, Kenema, Sierra Leone (M. Vandi)

**Keywords:** Ebola virus disease, survivors, sequelae, Sierra Leone, viruses

## Abstract

We rapidly assessed the health of Ebola virus disease (EVD) survivors in Kenema, Sierra Leone, by reviewing medical charts of all patients attending the Survivor Clinic of Kenema Government Hospital. Data were abstracted on signs and symptoms at every attendance. As of November 2015, a total of 621 attendances by 115 survivors with laboratory-confirmed EVD were made to the Survivor Clinic. Most (60.9%) survivors were women. Survivors’ median age was 28 years (range 0.25–70 years). Survivors attended the clinic a median of 5 times (range 1–21 times) each, and the median time from EVD discharge to attendance was 261 days (range 4–504 days). The most commonly reported signs and symptoms among the 621 attendances were headache (63.1%), fever (61.7%), and myalgia (43.3%). Because health needs of EVD survivors are complex, rapid chart reviews at survivor clinics should be repeated regularly to assess the extent of illness and prioritize service delivery.

After Ebola virus disease (EVD) emerged in Sierra Leone in 2014, a total of 8,704 confirmed cases and 3,955 deaths had been reported as of the declaration of EVD transmission-free status on November 7, 2015 (*1*). Approximately 4,000 survivors are in the country, and evidence of the frequency and duration of sequelae over an extended period is limited in this cohort.

Recently published studies highlight the occurrence of post-EVD complications, such as uveitis (*2*,*3*) and encephalopathy (*4*), but these are isolated investigations. Evidence on the prevalence of these and other sequelae is increasing. There is a growing body of evidence on the burden of these and other sequelae, including 1 report based on a survey of 81 survivors in Kenema (Sierra Leone), which found that nonspecific symptoms, such as arthralgia, headache, and myalgia, persist for months after recovery from acute EVD (*5*–*7*). Many survivors also face EVD-related stigma and rejection from their communities (*5*,*8*) and suffer with posttraumatic stress disorder, depression, or anxiety (*5*,*7*–*9*). Furthermore, new ocular problems, such as uveitis, are commonly reported among survivors (*2*,*5*,*6*,*10*). A summary of the literature on these sequelae was presented in a recently published review article, but evidence from the 2014–15 West Africa outbreak had a maximum duration of follow-up of 1 year (*11*).

To rapidly assess the health of survivors in Kenema District, we reviewed the medical charts of all patients of the Survivor Clinic at Kenema Government Hospital (KGH) to determine the frequency of possible EVD-related sequelae among persons in this cohort, many of whom had been in convalescence for >1 year. Secondarily, we determined the frequency of diseases diagnosed within this group.

## Materials and Methods

### Setting

KGH is the major Ministry of Health and Sanitation, Government of Sierra Leone, referral hospital in Kenema District, the third most populous district in Sierra Leone (population 609,873 [Sierra Leone Population and Household Census 2015, https://www.statistics.sl/wp-content/uploads/2016/06/2015-Census-Provisional-Result.pdf]). In October 2014, an EVD Survivor Clinic was opened at KGH to provide treatment and care at no cost to survivors. This nurse-led clinic has support from an on-call medical doctor and provides treatment for minor complaints and mental health counseling. The Ministry of Social Welfare, Gender and Children’s Affairs of the Government of Sierra Leone maintains a register of EVD survivors in Kenema to facilitate the provision of benefits from the government and nongovernmental organization partners. As of November 2015, records from the Ministry of Social Welfare, Gender and Children’s Affairs in Kenema indicated that 162 EVD survivors were registered to receive benefits there.

### Data Entry

After a rapid review of the literature, we developed an electronic proforma to collect demographic data and signs and symptoms previously reported as EVD sequelae using Microsoft Excel 2010 (Microsoft Corp., Redmond, WA, USA) (*5*). A close-ended list of signs and symptoms was used for consistent data collection. This list included fever because signs and symptoms reported with fever might indicate other acute infectious diseases, rather than EVD sequelae. Data were then abstracted from individual hard copy medical charts for all patients who attended the Survivor Clinic during November 10–13, 2015, by a medical doctor (A.O.V.) and entered by the lead author (H.M.).

For each patient, we collected name, sex, age, address, and date of discharge from KGH after the diagnosis and treatment of EVD and for each of their attendances during convalescence, their complaints (signs and symptoms), and differential diagnoses. Each sign and symptom was collected as a binary variable (yes/no), and the differential diagnoses were entered as open-ended text (verbatim from the medical chart); this text was later queried to generate a list of diseases and conditions by searching for selected text. After entry, a unique identifier was generated for each patient, and personally identifiable information was stripped from the dataset and stored separately on a password-protected terminal at the World Health Organization in Kenema. These patients’ identities were then cross-referenced against the national viral hemorrhagic fever database developed by the US Centers for Disease Control and Prevention (Atlanta, GA, USA) (*12*) and maintained by the Kenema District Health Medical Team EVD Response Centre to confirm these patients had laboratory-confirmed EVD.

### Data Analysis

Data analysis was performed on the anonymized dataset and restricted to patients with confirmed EVD. We calculated the time (days) from EVD discharge to attendance date and determined attendance-level frequencies of signs and symptoms (first among all attendances, then among attendances during which fever was not reported). To determine which signs and symptoms were associated with febrile presentation, we assessed associations between each and fever (yes/no) using univariate generalized estimating equations (GEE) logistic regression to account for the clustering of attendances by patient. We also determined associations between fever and age (quartiles), sex, and time since EVD discharge (quartiles) using univariate GEE logistic regression. Similarly, to determine whether signs and symptoms varied between children (<18 years of age) and adults (>18 years of age), we determined associations between age and each sign and symptom using univariate GEE logistic regression, then in GEE models adjusted for the time since discharge. All data cleaning, management and analysis were performed by using Stata version 13.1 (StataCorp LP, College Station, TX, USA). We considered p values <0.05 to be statistically significant.

### Ethics Statement

This review of medical charts was conducted as part of the response to the outbreak of EVD, a public health emergency of international concern (*13*). Patient consent was not sought because we performed a secondary analysis of medical chart data. We obtained approval for the review from the District Medical Officer (Ministry of Health & Sanitation).

## Results

### Description of Survivors

From the opening of the Survivor Clinic in October 2014 until November 13, 2015, a total of 124 survivors sought care at the clinic. Most (115 [92.7%]) previously had laboratory-confirmed EVD, and these 115 patients had attended the clinic a total of 621 times.

 Most of the confirmed EVD survivors were women (70 [60.9%] of 115) and residents of Kenema District (109 [94.8%]), among whom most (96 [88.1%] of 109) were residents of Nongowa Chiefdom, from which most EVD cases in the District were reported during the outbreak. The median patient age was 28 (range 3 months to 70 years).

**Figure 1:**
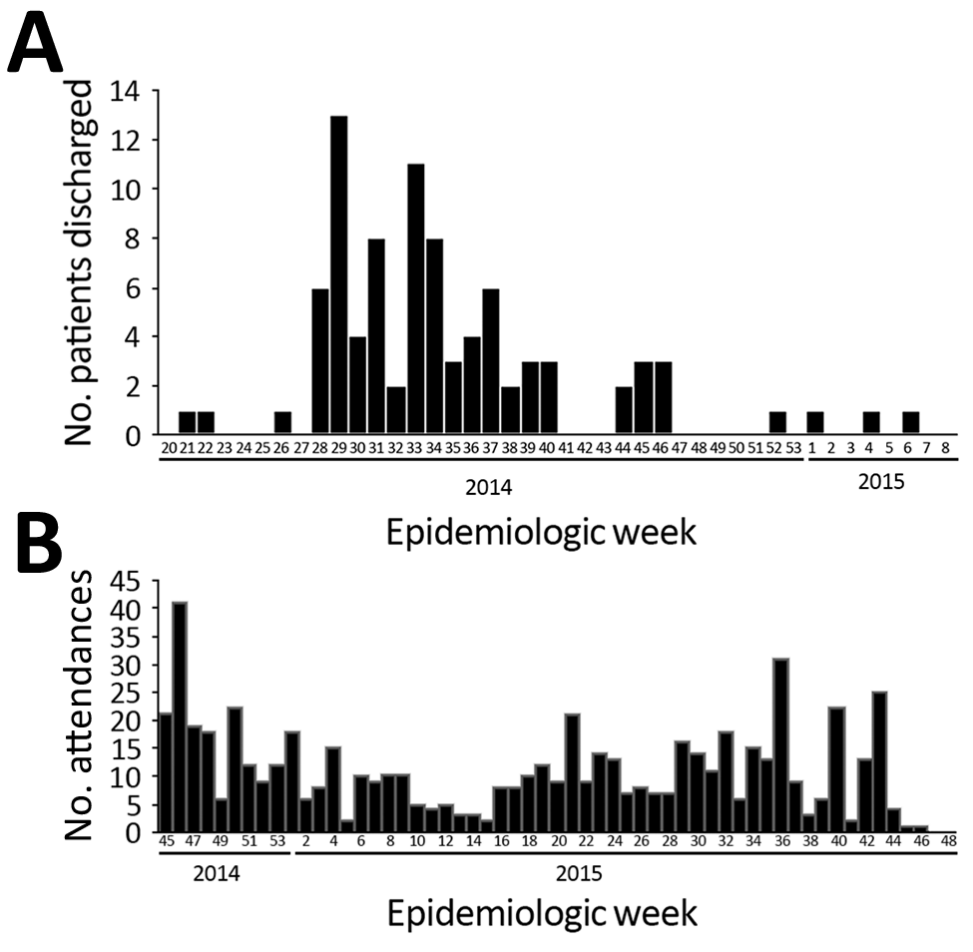
Hospital discharge and attendances for 115 survivors of laboratory-confirmed Ebola virus disease (EVD) attending the Survivor Clinic at Kenema Government Hospital (KGH), Kenema, Sierra Leone), 2014–2015. A) Discharge from KGH after initial EVD diagnosis for the 88 (76.5%) survivors for whom data were available on date of EVD discharge. B) Dates of the 621 attendances at KGH during convalescence by the 115 EVD survivors.

Patients attended the clinic a median of 5 times (range 1–21 times). Median time from EVD discharge to first attendance was 114 days (4–395 days) and from EVD discharge to any attendance was 261 days (4–504 days) (Figure 1).

### Signs and Symptoms

**Figure 2:**
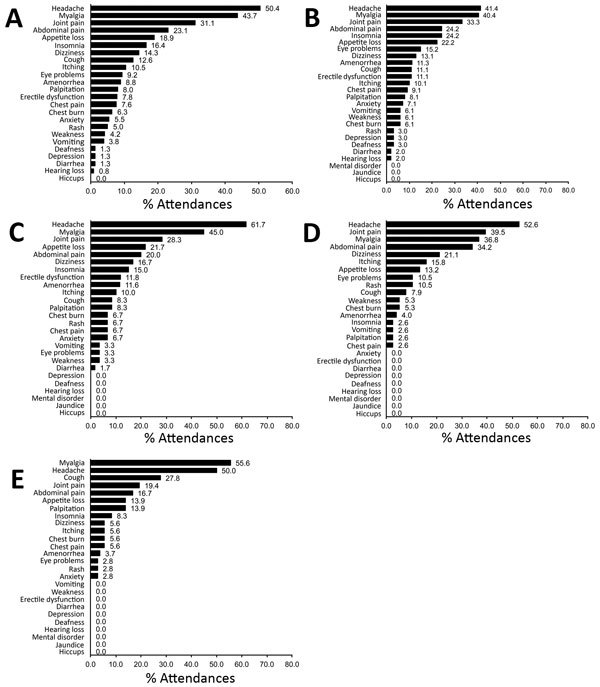
Frequency of symptoms reported without fever at 238 attendances of survivors of laboratory-confirmed Ebola virus disease (EVD) at the Survivor Clinic, Kenema Government Hospital (KGH), Kenema, Sierra Leone, 2014–2015. A) Overall; B) 4–144 days from KGH discharge after initial EVD diagnosis to Survivor Clinic attendance; C) 145–254 days from discharge to attendance; D) 255–358 days from discharge to attendance; and E) 359–504 days from discharge to attendance. Eye problems comprise eye irritation, eye pain, eye discharge, itchy eye, poor vision, or blurred vision. Amenorrhea was recorded only for women (age range 15–40 years) and erectile dysfunction only for men (age range 24–35 years). Chest burn is a local term for heartburn.

Of the 621 attendances by the 115 survivors, the most commonly reported symptoms were headache (63.1% [95% CI 59.2%–67.0%]), fever (61.7% [95% CI 57.7%–65.5%]), and myalgia (43.3% [95% CI 39.4%–47.3%]). Joint pain was the fifth most common symptom (30.1% [95% CI 26.5%–33.9%]). At the 238 attendances for which patients did not have fever, the most commonly reported symptoms were headache (50.4%), myalgia (43.7%), and joint pain (31.1%) (Figure 2, panel A), but the relative frequencies changed with increasing time since EVD discharge (Figure 2, panels B–E).

At least 1 eye problem was reported at 51 attendances; the most common of these were blurred vision (37.3%), poor vision (29.4%), and itchy eyes (25.5%). When we considered time since EVD discharge, eye problems were most commonly reported within the first quartile of follow-up (4–144 days after discharge; Figure 2, panels B–D). However, these eye problems also were reported much later; the median time from EVD discharge to attendance with an eye problem was 146 days (range 35–380 days).

Referrals to mental health counselling were reported only at 4 attendances by 4 unique survivors, of whom 2 were reported with depression: 1 occurred 146 days and the other 172 days after EVD discharge. However, based on feedback from clinic staff, these mental health referrals were not consistently reported in the medical charts; thus, this figure underestimates the rate of referrals.

Of the 115 survivors, 33 (28.7%) were <18 years of age. Compared with attendances by survivors >18 years of age (n = 489), attendances by survivors <18 years of age (n = 131) (age was missing for 1 survivor) were significantly more likely to be reported with fever (72.5% vs. 58.7%, p = 0.015), coughing (42.8% vs. 29.5%, p = 0.015), rash (14.5% vs. 2.7%, p<0.001), and vomiting (16.8% vs. 6.3%, p = 0.002) but less likely to be reported with myalgia (30.5% vs. 46.8%, p = 0.001) and insomnia (7.6% vs. 16.2%, p = 0.009). Survivors <18 years of age were also less likely than those >18 years of age to have been reported with eye problems (5.3% vs. 9.0%), but this finding was not statistically significant (p = 0.255). We found no other statistically significant differences in symptoms between survivors <18 years and >18 years of age, and all associations remained similar in magnitude and significance after adjustment for time since discharge (data not shown).

**Figure 3:**
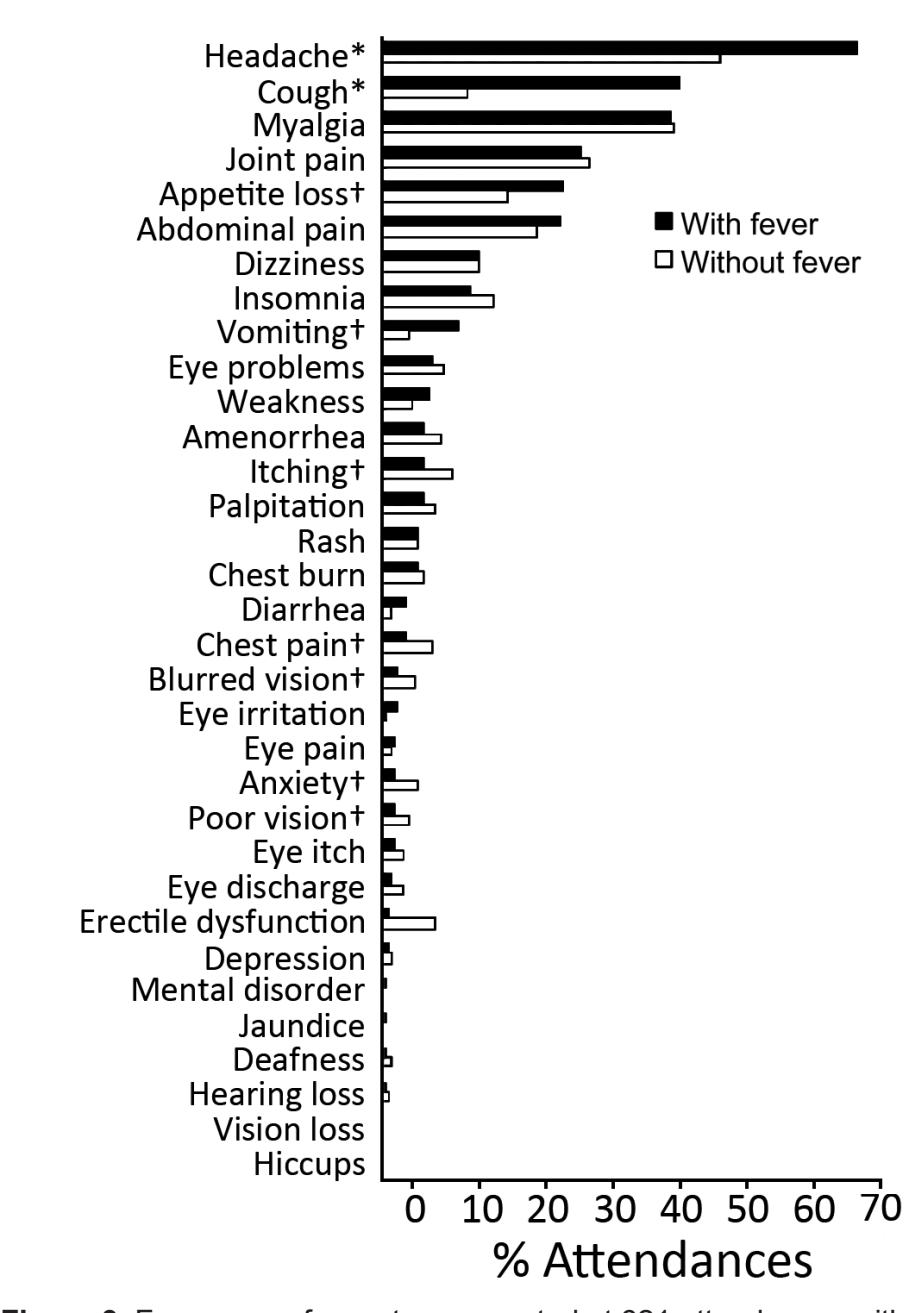
Frequency of symptoms reported at 621 attendances with and without reported fever by 115 survivors of laboratory-confirmed Ebola virus disease at the Survivor Clinic, Kenema Government Hospital, Kenema, Sierra Leone, 2014–2015. Fever was recorded for 61.7% total attendances. Amenorrhea was recorded only for women (age range 15–40 years) and erectile dysfunction only for men (age range 24–35 years). Chest burn is a local term for heartburn. p values are from univariate generalized estimating equations logistic regression analysis. *p<0.01; †p<0.05.

When comparing attendances reported with (n = 383) or without (n = 238) fever (Figure 3; Table), we found that cough (p<0.001), headache (p<0.001), vomiting (p = 0.010), and appetite loss (p = 0.028) were significantly more likely to be reported with fever. Itching (p = 0.028), blurred vision (p = 0.034), poor vision (p = 0.043), chest pain (p = 0.044), and anxiety (p = 0.014) were significantly less likely to be reported with fever. Fever was also significantly less likely to be reported at attendances closer to the date of EVD discharge (4–144 days [p<0.001] and 145–254 days [p<0.001] vs. 359–504 days after EVD discharge). Similarly, survivors 29–35 (p = 0.025) and 36–70 years of age (p = 0.010) were significantly less likely than survivors <18 years of age to have fever.

**Table:** Characteristics reported at 621 attendances by 115 survivors of laboratory-confirmed Ebola virus disease attending the Survivor Clinic at Kenema Government Hospital, Sierra Leone, 2014—2015*

Characteristic	Overall†	Fever‡		p value§
		Yes	No	
Sex				
M	187 (30.7) [27.1–34.4]	123 (30.2)	64 (25.1)	0.309
F	423 (69.3) [65.6–72.9]	284 (69.8)	191 (74.9)	Ref
Duration since discharge, d				
4–144	131 (21.1) [18.1–24.5]	61 (14.8)	107 (42.1)	<0.001
145–254	134 (21.6) [18.5–25.0]	96 (23.4)	69 (27.2)	<0.001
255–358	221 (35.6) [31.9–39.5]	125 (30.4)	41 (16.1)	0.321
359–504	134 (21.6) [18.5–25.0]	129 (31.4)	37 (14.6)	Ref
Age, y				
<18	153 (25.0) [21.7–28.5]	109 (26.4)	39 (15.1)	Ref
18–28	148 (24.1) [20.9–27.7]	86 (20.8)	52 (20.1)	0.178
29–35	153 (25.0) [21.7–28.5]	142 (34.4)	110 (42.5)	0.025
36–70	159 (25.9) [22.6–29.6]	76 (18.4)	58 (22.4)	0.010
Median (range)	30.0 (0.25–70.00)	30.0 (0.25–70.00)	33.0 (3–70.00)	0.001
Signs and symptoms¶				
Joint pain	187 (30.1) [26.6–33.8]	113 (29.5)	74 (31.1)	0.627
Myalgia	269 (43.3) [39.5–47.3]	165 (43.1)	104 (43.7)	0.874
Cough	201 (32.4) [28.8–36.2]	171 (44.7)	30 (12.6)	<0.001
Weakness	37 (6.0) [4.3–8.1]	27 (7.1)	10 (4.2)	0.097
Dizziness	89 (14.3) [11.8–17.3]	55 (14.4)	34 (14.3)	0.915
Rash	32 (5.2) [3.7–7.2]	20 (5.2)	12 (5.0)	0.903
Itching	49 (7.9) [6.0–10.3]	24 (6.3)	25 (10.5)	0.028
Blurred vision	19 (3.1) [2.0–4.8]	8 (2.1)	11 (4.6)	0.034
Vision loss	0	0	0	NA
Eye discharge	12 (1.9) [1.1–3.4]	5 (1.3)	7 (2.9)	0.097
Poor vision	15 (2.4) [1.5–4.0]	6 (1.6)	9 (3.8)	0.043
Eye pain	10 (1.6) [0.9–3.0]	7 (1.8)	3 (1.3)	0.837
Eye itch	13 (2.1) [1.2–3.6]	6 (1.6)	7 (2.9)	0.113
Eye irritation	9 (1.4) [0.8–2.8]	8 (2.1)	1 (0.4)	0.119
Eye problems	51 (8.2) [6.3–10.7]	29 (7.6)	22 (9.2)	0.205
Headache	392 (63.1) [59.2–66.8]	272 (71.0)	120 (50.4)	<0.001
Diarrhea	17 (2.7) [1.7–4.4]	14 (3.7)	3 (1.3)	0.116
Vomiting	53 (8.5) [6.6–11.0]	44 (11.5)	9 (3.8)	0.010
Insomnia	89 (14.3) [11.8–17.3]	50 (13.1)	39 (16.4)	0.370
Chest burn	35 (5.6) [4.1–7.8]	20 (5.2)	15 (6.3)	0.562
Chest pain	31 (5.0) [3.5–7.0]	13 (3.4)	18 (7.6)	0.044
Abdominal pain	157 (25.3) [22.0–28.9]	102 (26.6)	55 (23.1)	0.620
Appetite loss	149 (24.0) [20.8–27.5]	104 (27.2)	45 (18.9)	0.028
Depression	6 (1.0) [0.4–2.1]	3 (0.8)	3 (1.3)	0.759
Anxiety	20 (3.2) [2.1–4.9]	7 (1.8)	13 (5.5)	0.014
Mental disorder	2 (0.3) [0.1–1.3]	2 (0.5)	0	NA
Palpitation	42 (6.8) [5.0–9.0]	23 (6.0)	19 (8.0)	0.223
Jaundice	1 (0.2) [0.0–1.1]	1 (0.3)	0	NA
Hiccups	0	0	0	NA
Deafness	4 (0.6) [0.2–1.7]	1 (0.3)	3 (1.3)	0.218
Hearing loss	3 (0.5) [0.2–1.5]	1 (0.3)	2 (0.8)	0.350
Amenorrhea	31 (7.3) [5.2–10.2]	16 (6.3)	15 (8.8)	0.222
Erectile dysfunction	6 (3.2) [1.4–7.0]	1 (0.8)	5 (7.8)	0.047

*Within each cross-tabulation, because of missing data, the sum of the component cells may be less than the total number of attendances. Where a zero cell was reported in the cross-tabulation with fever, no p value is reported. NA, not applicable; Ref, reference category. †All values are no. (%) [95% CI] unless indicated otherwise indicated. ‡All values are no. (%) unless otherwise indicated. §p values are from univariate generalized estimating equations logistic regression analysis. ¶ Amenorrhea was recorded only for women (age range 15–40 years) and erectile dysfunction only for men (age range 24–35 years). Chest burn is a local term for heartburn.

**Figure 4:**
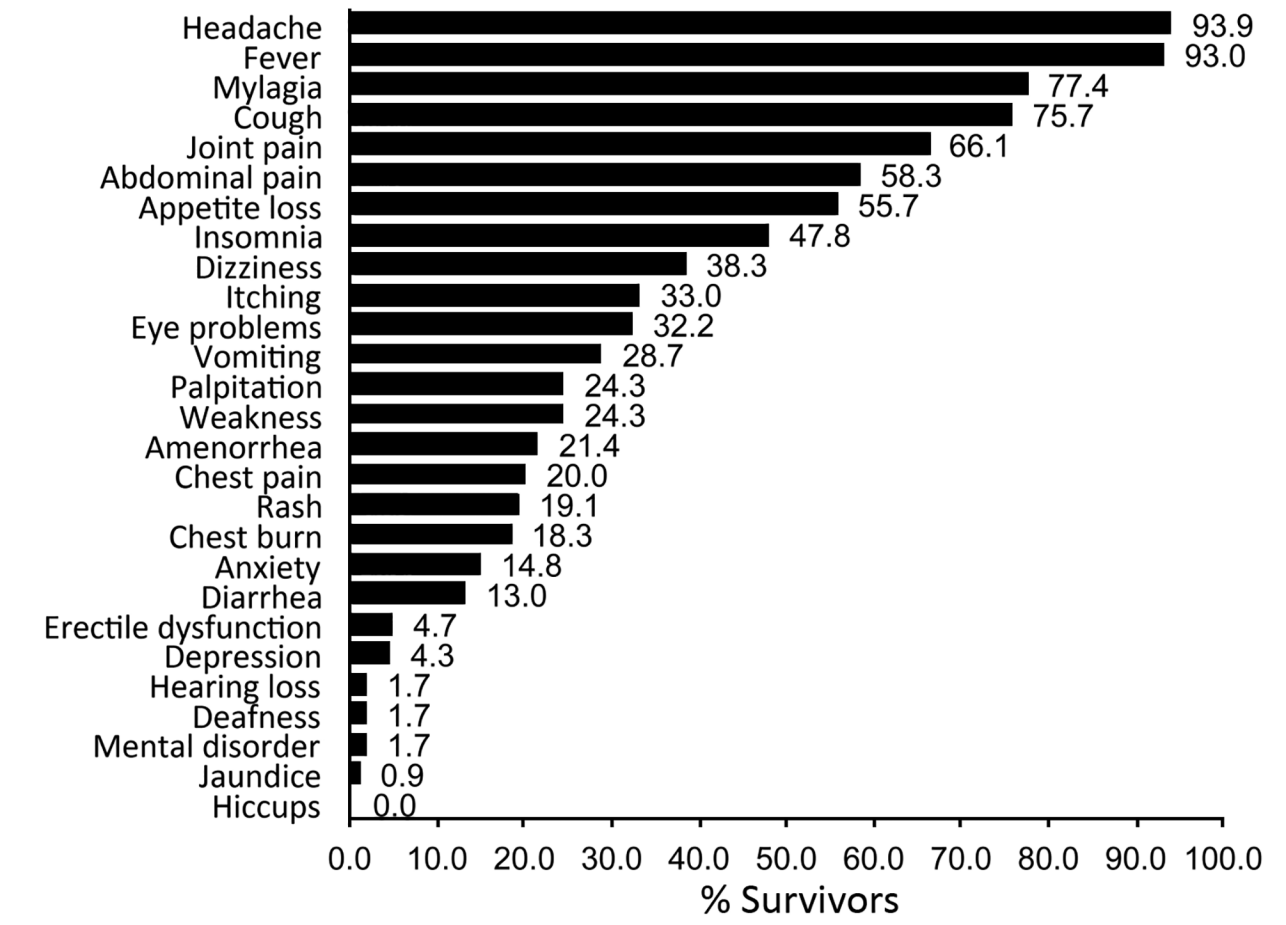
Frequency of symptoms reported by 115 survivors of laboratory-confirmed Ebola virus disease attending the Survivor Clinic, Kenema Government Hospital, Sierra Leone, 2014–2015. Eye problems comprise eye irritation, eye pain, eye discharge, itchy eye, poor vision, or blurred vision. Amenorrhea was recorded only for women (age range 15–40 years) and erectile dysfunction only for men (age range 24–35 years). Chest burn is a local term for heartburn.

When we determined the frequency of symptoms for the 115 survivors over their entire attendance history (Figure 4), we found the most commonly reported symptoms were headache (93.9% [95% CI 87.9%–97.5%]), fever (93.0% [95% CI 86.8%–96.9%]), and myalgia (77.4% [95% CI 68.7%–84.7%]); eye problems were reported by 37 (32.2% [95% CI 23.8%–41.5%]) survivors. All but 8 survivors were reported as having had fever at least once, so frequencies of symptoms stratified by the presence or absence of fever are not presented.

### Morbidity

**Figure 5:**
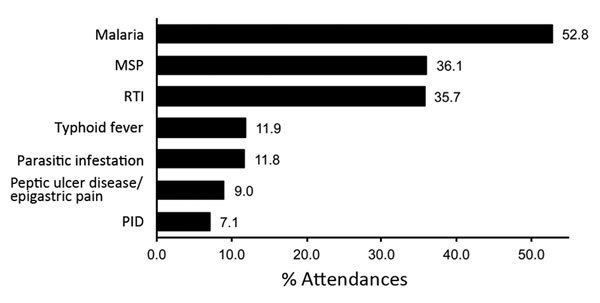
Diseases and conditions with at least a 5% frequency diagnosed at 621 attendances by 115 survivors of laboratory-confirmed Ebola virus disease at the Survivor Clinic at Kenema Government Hospital, Kenema, Sierra Leone, 2014–2015. MSP, musculoskeletal pain; PID, pelvic inflammatory disease (women only); RTI, respiratory tract infection.

Of the 621 attendances by the 115 survivors, the most commonly listed differential diagnoses were malaria (52.8% [95% CI 48.8%–56.8%]), musculoskeletal pain (36.1% [95% CI 32.3%–40.0%]), and respiratory tract infections (23.2% [95% CI 19.9%–26.7%]) (Figure 5). However, malaria was not routinely confirmed through diagnostic testing and often was diagnosed on the basis of clinical presentation.

For 45 (7.2%) attendances, musculoskeletal pain was given as the differential diagnosis either solely or in the presence of 1 of the following: abscess, diarrhea, erectile dysfunction, arthritis/sore throat, insomnia, “post-Ebola weakness,” scabies, or ulcer. For the 31 attendances for which only musculoskeletal pain was diagnosed in the absence of fever, the median time from EVD discharge was 231 days (range 43–464 days).

## Discussion

Our comprehensive review of the medical records of patients attending the Survivor Clinic at KGH revealed that many experienced potential EVD-related sequelae >1 year after hospital discharge. The most commonly reported sequelae at afebrile presentation were headache, myalgia, joint pain, and abdominal pain, which is largely consistent with reports from survivors of the 2013–15 West Africa outbreak (*5*,*8*,*14*,*15*). A retrospective cohort study of EVD survivors and their contacts in Uganda highlighted that long-term sequelae persist for >2 years (*10*), but similar evidence is lacking from the West Africa EVD outbreak, which was caused by a different virus species (Zaire).

Nanyonga et al. reported frequent sequelae at least 4 months after discharge among survivors in Kenema and, because their survey was based on a cross-sectional study of a small convenience sample of survivors, recommended a more systematic assessment of sequelae (*5*). Accordingly, our report is based on an analysis of a longitudinal dataset of all attendances at the KGH Survivor Clinic over a longer period of follow up; thus, it contributes to the understanding of the patterns of sequelae within this cohort. Our findings are consistent with those of the survey by Nanyonga et al. (*5*), which reported joint pain, headache, and myalgia as the most common sequelae, and those of another review of data from medical consultations at a survivor clinic in Freetown, Sierra Leone; in the case of the latter, arthralgia, fatigue, and abdominal pain were the 3 most common complaints (*15*). In the Freetown report, data were reported from presentations earlier in the course of convalescence than in our review, and no distinction was made on the basis of afebrile or febrile status, but the authors were able to evaluate risk factors for uveitis.

New ocular symptoms were reported from >55% of persons attending survivor clinics in Port Loko and Freetown (Sierra Leone), and the incidence of uveitis was high (*6*,*15*). In comparison, in our analysis, the frequency of eye problems was lower, but this finding may be due to underreporting because survivors were not assessed by ophthalmologists at the KGH Survivor Clinic. Also, although eye problems were documented in the medical charts, the frequency of uveitis could not be determined because slit-lamp examinations were not provided at the KGH clinic. In our chart review, >90% of the survivors were reported as having had fever at least once since being discharged; although no information is available about the etiology, transient fevers have been reported in a few survivors months after recovery, suggesting that fever might be an underreported sequela of EVD (*11*). Although reports of EVD recrudescence (*16*) are limited, given the high frequency of febrile illness among the survivors at KGH, further research is needed to assess the persistence of Ebola virus in immunologically protected body sites to inform guidelines on retesting.

Our findings suggest that survivors’ needs vary with age. Survivors <18 years of age sought care for complaints that differed from those of adults. This finding was reported previously in Freetown; that report, despite using a different age cutoff, also found that persons <16 years of age were more likely than those >16 years of age to have rash and were less likely to have insomnia (*15*).

This medical chart review captured data on all attendances by all confirmed EVD survivors attending the Survivor Clinic. We cannot definitively state that symptoms such as myalgia and joint pain did not result from infection with other pathogens endemic to the community, but we were able to assess the frequency of symptoms in the absence of fever. Itching, blurred vision, poor vision, chest pain, and anxiety were more likely to be reported among attendees without fever than with fever. Furthermore, survivors attended the clinic up to 464 days after EVD discharge with the sole complaint or musculoskeletal pain, providing evidence of the persistence of this sequela >1 year after EVD discharge.

Malaria was diagnosed at ≈50% of attendances, but because we restricted our review to EVD survivors, we cannot infer a higher incidence of malaria or of other illnesses in the absence of a comparison group, especially given the estimated increased malaria incidence during the EVD outbreak (*17*). Because many diseases were diagnosed without laboratory confirmation, illnesses might have some degree of misclassification. Most patients attending the KGH Survivor Clinic resided in Nongowa Chiefdom, where KGH is located; EVD survivors from other chiefdoms might be less likely to attend KGH because of the cost and difficulty of transport.

Our review has some limitations because the medical charts did not consistently document the number or outcome of any pregnancies in female survivors or referrals to mental health counseling. Counseling sessions were performed at the Survivor Clinic, so they might have been performed as part of the clinic visit without being documented in the medical chart. Additionally, because our review considered only live survivors, we do not have information about deaths during convalescence due to EVD (*15*), EVD-related sequelae, or non-EVD causes. We were able to assess only the presence of signs and symptoms, and no information was available about their severity. Because our exploratory analysis was to assess the association between each of 33 signs and symptoms with febrile presentation, some associations we found might have been due to chance. Last, our review used only data contained within the medical charts; thus, we have no information about the Ebola virus viral load of survivors while they had acute EVD. Although recently published reports suggest that higher viral loads are associated with specific sequelae, such as headache or uveitis, we could not assess this (*6*,*14*).

Given the large cohort of EVD survivors after the West Africa outbreak, a more universal assessment of possible sequelae should be performed in other districts of Sierra Leone. Using district-level registers of survivors, a prospective age, sex and district-matched cohort study could quantify the risk for EVD sequelae and other illnesses. Because this outbreak has ended in the 3 most affected countries and because the health needs of survivors are complex and not yet fully understood (*11*), the risk exists that sustained essential services might not be available for EVD survivors in the region. Rapid chart reviews at survivor clinics should be repeated at regular intervals to review the persistence of sequelae and the incidence of illness, which can, in turn, be used to prioritize service delivery.
